# Perception of Vowel Segments by Children and Adults: A Preliminary Study of Segment Duration

**DOI:** 10.3390/brainsci16090992

**Published:** 2026-09-19

**Authors:** Mark Hedrick, Kelly Yeager, Cary Springer

**Affiliations:** 1 Department of Audiology & Speech Pathology, The University of Tennessee Health Science Center, Knoxville, TN 37996, USA; kyeager@uthsc.edu; 2 Office of Innovative Technologies, The University of Tennessee, Knoxville, TN 37996, USA; springer@utk.edu

**Keywords:** vowel perception, speech perception, children’s perception

## Abstract

**Highlights:**

**What are the main findings?**

- Our results show that differences between children’s (aged 4 to 9 years) and adults’ perception of vowels may arise from children being more dependent on change/contrast than adults to identify a vowel.
- Children aged 4 to 9 years needed more of an acoustic change/contrast than adults to identify a vowel.
- There was not a significant effect of vowel context for children and adults for corner vowels.

**What are the implications of the main findings?**

- Perceptual differences between children aged 4 to 9 and adults may be tied to children’s dependence on acoustic change and contrast.
- Children may need more of an acoustic change/contrast than adults to make vowel judgments.
- Children aged 4 to 9 years may not be as dependent upon coarticulation for accurate perception of corner vowels.

**Abstract:**

**Background/Objectives**: Children’s perception of phonemes differs from that of adults. Our purpose was to examine whether children’s perception of vowels differs from that of adults because of differential emphasis on vowel segments (steady-state or transition), degree of coarticulation, or amount of information to make a perceptual judgment of the vowel. **Methods**: A, between-group cross-sectional design was employed. Twenty-four children aged four to nine years and 17 adults (aged 18 to 53) participated. Stimuli were three naturally produced syllables (/bɑ/, /bi/ and /bu/), presented in the sound field in entirety or segmented to isolate the formant transition or the vowel static formant center. Participants selected the vowel presented by choosing from corresponding pictures on a computer screen. **Results**: The data from children showed consistently less accuracy for vowel identification than adults for whole syllable and all segments except full transitions. **Conclusions**: Children were more attuned to acoustic change or contrast to enable vowel perception than adults, yet needed more extensive change before reaching adult performance.

## 1. Introduction

Vowel perception is important for accurate word and sentence recognition [1,2,3,4], and for delimiting lexical access by preschoolers [5,6]. Previous research into vowel perception led with the assumption that vowels were identified by idealized static target formant patterns that were independent of surrounding phonetic context. This interpretation of vowel perception came to be known as the simple target model [7]. Subsequent research findings, however, showed that the influence of phonetic environment and coarticulation prevented consistent static formant patterns for vowels [8], and that listeners could identify vowels by formant transitions into and from the steady-state portion of the vowel as accurately as from the steady-state part itself [9,10]. Taken together, then, both the trajectory and pattern of formants, as well as phonetic context and duration, affect the perceptual identity of the vowel [8,11]. It would appear that this information is combined across time to determine vowel identity [10,11,12]. Children with normal hearing can use both formant transitions and vowel steady-state formant patterns to identify vowels [13,14,15].

Earlier work showed that children even as old as eleven years may still not have fully developed adult-like internal rules and operations for phoneme perception [16]. With regard to vowel perception, there could be at least three developmental explanations for this discrepancy between children and adults: differential emphasis on vowel segments (whether steady-state or formant transition), degree of coarticulation, or that children need more acoustic information than adults to identify a given vowel. A number of studies have shown that children appear to attend more on larger units of speech, with dynamic cues (such as transitions) salient because they tie segments of speech together. With development, children will later focus on smaller units or smaller phonemic segments of speech [17,18,19,20,21,22]. Thus, children may not be able to efficiently focus on and use all segments of the vowel. A related explanation to that of formant transitions would be the degree of coarticulation that may be needed by children to accurately identify a vowel. For instance, with a consonant-vowel (CV) syllable having the bilabial /b/ as the initial consonant, the degree of coarticulation would likely be least for /bi/, greater for /bɑ/, and greatest for /bu/ [23,24]. For /bi/, the tongue is restricted, and thus less coarticulation. For /bɑ/, the tongue and jaw will move to a neutral position, thus relatively large degree of coarticulation. For /bu/, there is lip rounding as well as a back tongue placement, hence even more coarticulatory influence than for /bi/ or /bɑ/. Indeed, the degree of coarticulation in speech production for children aged three to seven years versus adults is significantly greater for the children for any labial-vowel combination [25]. This degree of coarticulation may then lead children to perceptually favor the information from the transition. Perhaps then it is the overall degree of coarticulation that is most salient for children [26]. An explanation with reference to amount of acoustic information would suggest that, because of continued maturation, children simply need more of a given cue (either in amplitude or duration) to encode and thereby enable correct vowel identification [15,27,28].

One way to provide evidence toward these explanations is to acoustically manipulate speech, infer what each hypothesis would predict, and then see how the results measure up with the predictions. One such acoustic manipulation would be vowel duration. It is known that adult listeners can accurately determine vowel identity from only one glottal cycle [29], a shorter duration than was required to extract a pitch percept from the same listeners. But what about children? Would they also show so readily vowel identification even for shorter vowels, or shorter segments of vowels?

Our purpose was to determine whether typically-developing children could identify shortened segments of cardinal vowels as readily as adults, particularly when only certain acoustic cues (e.g., transitions or static vowel centers) were present. We reasoned that the differential emphasis on vowel segments explanation would show more accurate identification of transitions by the children across all vowel contexts. The degree of coarticulation explanation would show greater accuracy based on degree of coarticulation, thus accuracy would proceed as follows: /bi/ < /bɑ/ < /bu/. An explanation based on children requiring more acoustic information would show that children simply need longer durations of segments than adults to accurately identify vowels, and thus the children would systematically show less accuracy for shorter segments irrespective of the type of cue or the degree of coarticulation.

## 2. Materials and Methods

### 2.1. Participants

Twenty-four children, aged four to nine years (mean six years, nine males), who were monolingual American English speakers with no learning, cognitive, or language deficits per parent report and negative clinical history were participants. The children’s data was reported in an earlier manuscript [30], in which the authors compared the typically-developing children’s responses to those from children who are deaf and hard of hearing. It should be noted that children in this age range (starting at around four years of age) show advantages in reasoning and decision-making, and show some advantages in perception and memory over older children [31]. Seventeen adults, aged 18 to 53 (mean 27 years, two males), also participated. All participants had measured hearing thresholds of 25 dB HL or better at 500, 1000, 2000, and 4000 Hz as indicated by a hearing screening completed prior to participation [32]. This research was approved by The University of Tennessee Health Science Center Institutional Review Board (IORG0000051).

### 2.2. Stimuli

All stimuli were modified from an earlier study [33], and the procedures were identical to that of a previous study [30]. The earlier study [30] reported on comparisons of typically-developing children versus children with sensorineural hearing loss. Data from the typically-developing children are reproduced in the current study along with novel, heretofore unreported data from adults to compare differences between the child and adult data. Sample size was based on the matching of children required for the earlier study. Three consonant-vowel syllables in a /b/-vowel context for the vowels /i/, /ɑ/, and /u/, were recorded by a male monolingual English speaker. A reason for using the vowels in the labial consonant context is the greater degree of lingual coarticulation [24]. The speech tokens were recorded in a sound-attenuated room with a Spher-O-Dyne laboratory microphone, held approximately one centimeter from the speaker’s mouth. The speaker uttered three to five syllables of a given CV syllable and then selected one of the middle productions as the stimulus. Productions were directed to a Tucker-Davis Technologies preamplifier (Model MA2, Tucker-Davis Technologies, Alachua, FL, USA), then routed to a 16-bit A/D converter (Model DD1, Tucker-Davis Technologies), and sampled at a rate of 12.5 kHz.

Using Adobe Audition version 1.5 (Adobe Systems Inc., San Jose, CA, USA), the syllables were segmented at zero crossings into five segments: the initial transition, one-half of the initial transition, the vowel, one-half of the vowel, and one-quarter of the vowel. Counting the entire syllable, then, this made for six tokens per vowel. To isolate the one-half and one-quarter segments, the midpoint of the segment was selected, then one-quarter or one-eighth of the total duration was selected on either side of the midpoint. All segments were made at zero crossings, which consequently resulted in segments being approximations. Selection of cuts were made based on visual inspection of spectrograms. For the transition, the cuts were made where it appeared that formant movement had ceased. Durations (in milliseconds) for each of the segments are as follows: for /bi/, whole 352, transition 68, half transition 37, vowel center 118, half vowel center 54, quarter vowel center 26; for (/bɑ/, whole 359, transition 89, half transition 40, vowel center 171, half vowel center 86, quarter vowel center 43; for /bu/, whole 401, transition 114, half transition 63, vowel center 137, vowel half center 68, vowel quarter center 44 ms.

Formant transition onset and vowel center frequency values (in Hz) for formants F1, F2, and F3 were as follows: for /bi/, F1 onset 318, center 256, F2 onset 2086, center 2514, F3 onset 2963, center 3295; for (/bɑ/, F1 onset 355, center 395, F2 onset 750, center 952, F3 onset 2576, center 2748; for /bu/, F1 onset 332, center 277, F2 onset 1181, center 1183, F3 onset 2666, center 2736.

Each syllable and related segments were matched up with a cartoon picture: The syllable /bi/ and its segments with a bumblebee, /bɑ/ and its segments with a sheep, and /bu/ and its segments with a ghost. On the computer screen, the bumblebee was located on the left side of the screen, the sheep in the center, and the ghost on the right. Picture order was consistent within and across participants. Representing each token with a picture helped to keepthe younger participants engaged in the task.

### 2.3. Procedure

Prior to participation, informed consent, and assent when applicable, was obtained. The task was explained to both the parent and child. All participants were told they would see three pictures on a computer screen and each picture ‘said something’. Further, participants were told “the bumblebee says ‘/bi/’”, “the sheep says ‘/bɑ/’”, and “the ghost says ‘/bu/’”. Participants were instructed to listen carefully for the word and either click on the picture that ‘said it’ or tell the experimenter which picture they chose.

Control of the computer mouse was given to adults and eight- to nine-year-olds. The younger participants were instructed to tell the experimenter their selection and point to it on the screen if the child forgot the picture label, they were asked to simply repeat the sound they heard. If the participant chose to repeat the word, the experimenter clicked on the corresponding picture for the participant since the goal was not to map the sound to the picture, but rather to accurately discriminate between vowel sounds.

### 2.4. Apparatus

Participants were seated in an 87.5” X 84” sound-attenuated booth facing a 24” Mac computer screen and an experimenter sat in a chair beside the participant. All stimuli were presented in the sound field at approximately 65 dB SPL through a Dell Zylux multimedia computer speaker in front of the participant. Stimulus presentations and data collection were conducted online through a custom SuperLab 4.5 program script (Cedrus Corporation, San Pedro, CA, USA).

### 2.5. Practice and Test Trials

A practice trial consisting of the three whole syllables was presented and the participant was asked to listen to the word and indicate their response. All tokens were presented seven times. Only the full production was presented, no stand-alone segments were included. The practice trial firstly ensured the participant was able to correctly identify the unmodified stimuli, with at least 80% overall accuracy, before continuing to test trials which were harder as they included the segments of each stimulus. The practice trials were primarily used to familiarize the subjects with the task, and to ensure that they understood the task. All children were able to meet practice criteria.

Participants then proceeded to experimental trials. Each participant was presented five blocks in which each stimulus was presented once per block, with random orderings of stimuli for each block. There was no time limit between presentation of the stimulus and subject response. Once a picture was selected, there was a 500 ms period of silence before the next stimulus presentation. Verbal encouragement was given regardless of response, but no feedback was given with regard to accuracy of response. Total testing time averaged approximately 25 min for each participant and all trials were randomized before the start of each testing session.

## 3. Results

Mean correct vowel responses across vowels and segments are presented in Table 1. The number of correct identifications for each stimulus was used as the dependent variable in a Generalized Estimating Equations (GEE) model with a binomial probability distribution and logit link function. The model included main effects for group, vowel type and segment as well as group by vowel and group by segment interactions. The three-way interaction and vowel by segment interactions were omitted to improve model stability. Post hoc contrasts were run comparing groups within each of level of significant interactions.

The results from the GEE model are shown in Table 2. There was a significant group by segment interaction. Vowel was not significant as a main effect or in the group by vowel interaction.

Results from post-hoc contrasts indicate that groups significantly differed for whole syllables (*p* = 0.034), half transitions (*p* < 0.001), vowel centers (*p* = 0.015), half vowel centers (*p* = 0.001) and one quarter vowel centers (*p* < 0.001) with adults getting significantly more correct than children. There were no significant group differences for full transitions (*p* = 0.110).

## 4. Discussion

Our results showed that, for adults, the task of vowel identification was easy, with no effects from vowel type or duration or type of vowel segment. Results from the children showed similar results to those of adults for full transition, but consistently showed less accuracy of vowel identification when transition was reduced in duration, or with vowel centers, or even with complete consonant-vowel syllables. There was no significant main effect of vowel, nor a significant group × vowel interaction. This consistent finding across all the vowel environments would be evidence for the idea that children were attending more to acoustic change or contrast than were the adults for vowel perception. Processes involved in differences between child and adult vowel identification could be peripheral encoding or sharpening of acuity, or executive cognitive functions such as attention. The current preliminary study cannot provide answers as to which of these influenced our results, although psychoacoustic data from children suggest that inattention may be a factor [34,35]. Since the vowels in the current study are corner vowels, or English vowels with the most extreme acoustic patterns, it seems unlikely that the results of the current study could be explained by broader auditory acuity. Because shortening the transition did result in children performing more poorly than adults, it would seem that children need more of an acoustic change or contrast to make adult-like judgments. The midpoint between full and half transitions for the stimuli was in the 50 to 80 ms range. For the current study, then, children needed at least this much or more of the transitions to secure vowel judgments equal to that of adults. This result would partly support the idea that children need more information before making accurate vowel judgments [15].

It was surprising that the children, while performing similar to that of adults for full transition segments, identified vowels in full syllable more poorly than that of adults. The prominence of transitions in influencing the children’s vowel perception in the current study is in agreement with earlier findings [18,19,20], although it should be mentioned that phoneme specific effects for weighting different cues has also been reported [21,36]. It has been suggested that children will initially focus on larger units of speech and only later in development focus on smaller units or smaller phonemic segments of speech [17,18,19,20,21,22]. Corroborating work [25] suggests that by the age of 3 children have moved from a holistic focus to being able to use individual segments of syllables. Data from the current study, however, show that children aged 4 to 9 may not be using whole syllables as well as a transition segment of the syllable. Perhaps the entire syllable, with relatively static portions, was not always dynamic enough for the children in the current study. It may be that acoustic change as opposed to holistic processing was the stronger influence on the perception of children in the current study.

The apparent inability of overall degree of coarticulation (or vowel context) to predict children’s vowel identification in the current study is in agreement with earlier findings [24] that a high degree of coarticulation did not translate to greater perceptual accuracy. Their results showed that although the /b/ consonant context showed large coarticulatory effects, it was the /d/ consonant context that showed better prediction of the following vowel. They explain this result by noting that changes in segment information from consonant to vowel occurs more rapidly for /d/ than for the extended-over-time coarticulation changes seen with /b/, similar to earlier postulations [36]. Such an abrupt change from consonant to vowel would seem to point to formant transitions as a likely perceptual cue. Perhaps in the current study and previous work [24], children need more of a consonant-to-vowel contrast or change before they are able to arrive at an accurate vowel percept. The lack of a significant effect of vowel context in the current study does not eliminate the effects of coarticulation on the results, but would seem to weaken this explanation for the perceptual results. Coarticulation was not independently manipulated in our stimuli, which limits the extent of our comment on the contribution of coarticulation in influencing the perceptual data. It may be that it is the acoustic dynamic change of transitions that is more influential in vowel perception rather than the transition acting as a coarticulatory bridge of consonant and vowel.

The response modality, that of adults and older children selecting for themselves the vowel versus younger children sometimes saying or pointing to the cartoon representing the vowel sound, may have influenced results in that the younger children may have pointed to what they felt most interesting or saying what next came to them rather than what they actually heard. The constant position of the cartoon figures could potentially have led to some response bias, and the cartoons were not counterbalanced in position to lessen confusion for the younger children. We did not systematically record how many children used which response modality. It is hoped that the blocking and random ordering of stimuli within the blocks could have minimized effects of response modality or of response bias.

The current preliminary study, with a limited number of subjects and of context, cannot definitively answer all questions regarding differences between adults’ and children’s perception of vowels. The current study represents differences between a heterogeneous child sample and adults, and is not of itself a clear developmental account. Increasing the variable space would possibly lend more cumulative evidence for the findings from the current study. At the same time, the limited variables investigated here, along with ceiling effects from the adults, didmake for some analysis difficulties. Thus, increasing variable space may add more evidence, but may not necessarily add more clarity to the findings.

## 5. Conclusions

This preliminary study provides evidence that children are more dependent upon the acoustic change/contrast of vowel segments, and that this influences their vowel perception when compared to the vowel perception of adults. Children also seem to need a more extensive acoustic change/contrast than adults to accurately identify vowels. It is left to future studies to elucidate the developmental processes and specific information used by children for their perception of vowels.

## Figures and Tables

**Table 1:** Mean correct vowel responses (out of 5 presentations) for children and adults across vowels and across segments (whole syllable, initial and ½ initial transition (TR), vowel center (VC), and ½ and ¼ vowel center). Standard deviations are in parentheses below the mean responses.

Vowel	Group	Whole	TR	½ TR	VC	½ VC	¼ VC
/i/	Children	4.75	4.67	4.67	4.83	4.71	4.46
		(0.61)	(0.76)	(0.64)	(0.48)	(0.62)	(1.06)
	Adults	5.0	4.70	5.0	4.88	5.0	5.0
		(0)	(0.47)	(0)	(0.48)	(0)	(0)
/ɑ/	Children	4.75	4.83	4.62	4.25	4.04	3.75
		(0.53)	(0.38)	(0.49)	(1.22)	(1.37)	(1.45)
	Adults	4.94	5.0	4.94	4.94	4.94	4.94
		(0.24)	(0)	(0.24)	(0.24)	(0.24)	(0.24)
/u/	Children	4.92	4.79	4.29	4.92	4.75	4.5
		(0.28)	(0.41)	(1.08)	(0.28)	(0.53)	(0.72)
	Adults	4.94	5.0	4.94	5.0	5.0	4.94
		(0.24)	(0)	(0.24)	(0)	(0)	(0.24)

**Table 2:** Results from the GEE model.

Source	Wald Chi Square	df	*p*
Segment	12.799	5	0.025
Vowel	2.147	2	0.342
Group	21.221	1	<0.001
Group × Segment	28.510	5	<0.001
Group × Vowel	3.375	2	0.185

## Data Availability

Data are available upon request from the authors.
